# Adaptive error compensation in CNC turning based on deep reinforcement learning and genetic algorithm fusion

**DOI:** 10.1038/s41598-026-57046-8

**Published:** 2026-06-11

**Authors:** Huaize Pan, Yuexia Lv, Wenfeng Bai

**Affiliations:** 1 School of Mechanical Engineering, Shandong Key Laboratory of CNC Machine Tool Functional Components, Qilu University of Technology (Shandong Academy of Sciences), Jinan, 250353 Shandong China; 2 Shandong Institute of Mechanical Design and Research, Jinan, 250031 Shandong China; 3 School of Intelligent Manufacturing, Huanghuai University, Zhumadian, 463000 Henan China

**Keywords:** CNC turning, Error compensation, Deep reinforcement learning, Genetic algorithm, Adaptive control, Precision manufacturing, Engineering, Mathematics and computing

## Abstract

Precision manufacturing in CNC turning operations faces persistent challenges from complex, time-varying error sources, encompassing thermal deformation, progressive tool wear, and force-induced deflections. In this work we propose an adaptive error compensation framework that brings deep reinforcement learning (DRL) and genetic algorithms (GA) into a coupled, bidirectional loop, rather than treating them as standalone tools. GA performs global hyperparameter and reward-weight optimisation, while DRL handles real-time adaptive control through continuous interaction with the cutting environment. Validation on aerospace-grade Ti-6Al-4 V turning yields a mean absolute error of 2.6 μm—an 86.3% compensation effectiveness—together with 38% faster convergence than standalone DRL and a process capability index of 1.67 that comfortably clears the six-sigma threshold. Beyond ablation against DRL-only and GA-only configurations, we benchmark the fusion algorithm against three mainstream predictive-compensation baselines (BPNN-based, LSTM-based, and PSO-based) under identical machining conditions, and the proposed approach retains a clear margin across MAE, RMSE, recovery time, and convergence. The limits of the method are also reported: compensation accuracy degrades once cutting parameters drift more than 30% beyond the training envelope or when the workpiece material differs substantially from the training distribution, a constraint that motivates the transfer-learning extensions discussed later in the paper.

## Introduction

Precision manufacturing in CNC turning operations remains a critical challenge in modern industry, since dimensional accuracy directly influences product quality and production efficiency. Over recent decades, machining error compensation has migrated from static offline correction toward dynamic adaptive strategies, yet persistent gaps in compensation accuracy and real-time adaptability continue to constrain manufacturing performance^1^. The underlying difficulty traces back to the complex, nonlinear, and time-varying nature of the error sources at play during turning—thermal deformation, progressive tool wear, machine vibration, and material heterogeneity^2^. Traditional compensation approaches, which mostly rely on empirical models or simplified analytical representations, struggle to capture these intricate interactions and so fail to deliver reliable performance under varying machining conditions^3^.

Before turning to reinforcement learning, it is worth grounding the discussion in what data-driven methods have actually achieved on machining accuracy prediction, and where they still fall short. Physics-informed and cross-domain monitoring schemes have recently shown that domain-discriminative generative networks, combined with empirical wear mechanisms, can mitigate the randomness of tool degradation under small-batch personalised machining; this represents real progress, but the resulting models stay tied to a single wear-type prediction task rather than closing the loop with on-line compensation^4^. Knowledge-embedded tool digital twins push further by predicting wear collaboratively across multiple tools and integrating multi-source sensor fusion, yet they still depend on offline KA-TCN inference and do not actuate corrections in real time^5^. Stacking-based ensemble learning has likewise delivered competitive accuracy for thermal-error prediction in feed systems, exploiting the heterogeneity of base learners to absorb temperature-deformation lags^6^. In parallel, spatio-temporal parallel networks with evolutionary mechanisms tackle the imbalance of wear samples under variable working conditions^7^, while anomaly recognition for high- and low-frequency sensing data exploits long short-term graph neural networks to filter coolant-spray and electromagnetic disturbances before they corrupt downstream models^8^. The pattern is unmistakable: each of these works has solved a meaningful slice of the prediction problem, but none of them simultaneously delivers (i) closed-loop compensation actuation, (ii) hyperparameter self-tuning over the operational life of the controller, and (iii) graceful behaviour when cutting conditions wander outside the training envelope. That triple gap is the one we set out to close.

Recent advances in artificial intelligence—deep reinforcement learning (DRL) in particular—have opened promising routes for tackling adaptive control problems in manufacturing. Unlike conventional methods that depend on explicit mathematical modelling, DRL lets the system learn compensation policies through direct interaction with the cutting environment^9^. Several pioneering studies have demonstrated the feasibility of applying DRL frameworks to machining error control, where the agent learns to adjust process parameters in response to real-time feedback signals^10^. The catch is that existing DRL-based compensation schemes suffer from slow convergence and poor sample efficiency, especially in the high-dimensional state spaces typical of multi-axis CNC systems^11^. The exploration-exploitation dilemma inherent to reinforcement learning further complicates the training process, often producing suboptimal policies that generalise poorly across diverse machining scenarios^12^.

Genetic algorithms (GA), as robust global optimisation techniques inspired by natural evolution, have a different but complementary record on complex engineering optimisation problems^13^. Within machining error compensation, GA has been applied to optimise compensation parameters and to identify favourable machining conditions, showing real strengths in multi-objective tasks over non-convex solution spaces^14^. Pure GA-based approaches, however, expose limitations of their own: their local search efficiency is modest and their computational overhead becomes uncomfortable when real-time compensation is required in dynamic environments^15^. The population-based evolutionary mechanism remains effective for global exploration but lacks the fine-tuning capability needed for precise error correction at the micro-scale^16^.

Despite substantial progress in both DRL and GA domains, current research predominantly treats these methodologies as independent tools rather than synergistic components. This separation overlooks potential complementary advantages: DRL excels in sequential decision-making and adaptive learning yet struggles with exploration efficiency; GA demonstrates strong global search capacity but lacks mechanisms for temporal dependency modelling^17^. The absence of integrated frameworks that combine the adaptive learning capability of DRL with the optimisation efficiency of GA represents a significant gap. Most reported hybrid approaches merely apply GA for hyperparameter tuning in DRL networks, without fundamentally addressing the core challenges of compensation precision, adaptation speed, and system robustness under variable operating conditions.

Several critical issues persist in contemporary error compensation research. First, compensation accuracy remains insufficient when confronting compound errors arising from multiple simultaneous disturbances. Second, adaptive capability proves inadequate for responding to abrupt changes in machining conditions, such as sudden tool wear or material property variations. Third, optimisation speed fails to meet real-time industrial requirements, particularly during initial learning phases when extensive exploration is necessary. These limitations collectively hinder the practical deployment of intelligent compensation systems in production environments, where reliability and consistency are paramount.

Addressing these challenges calls for a compensation framework that genuinely synthesises the strengths of DRL and GA, rather than chaining them sequentially. This research proposes an integrated methodology in which GA enhances DRL exploration through population-based experience generation, while DRL feeds gradient information back to guide GA evolution toward regions of higher compensation effectiveness. Such a fusion accelerates convergence, improves compensation precision, and strengthens adaptive responsiveness to dynamic machining conditions. Beyond theoretical contributions, the work aims to deliver practical solutions for industrial CNC turning operations, where enhanced dimensional accuracy translates directly into measurable productivity gains.

This paper presents three primary innovations. First, we establish a hybrid DRL-GA architecture specifically tailored for CNC turning error compensation, featuring bidirectional information exchange mechanisms that enable continuous mutual enhancement between learning and evolution processes. Second, we develop an adaptive compensation strategy that dynamically adjusts both the neural network policy and the genetic population parameters based on real-time error characteristics, achieving superior performance across varying operational regimes. Third, we design a multi-scale error modelling approach that decomposes complex machining errors into distinct components addressable through different optimisation pathways, improving computational efficiency without sacrificing accuracy. The subsequent sections elaborate on the proposed methodology, the experimental validation, and the comparative analysis against state-of-the-art compensation techniques.

## Theoretical foundation of CNC turning machining errors

### Analysis of error sources in CNC turning machining

Machining errors in CNC turning operations arise from multiple interacting sources, each contributing distinct characteristics to the overall dimensional deviation. Geometric errors, which stem from imperfections in machine tool construction and kinematic-chain transmission, form the primary static component^18^. These errors manifest through straightness deviations, perpendicularity misalignments, and positioning inaccuracies along motion axes. The geometric error vector at an arbitrary cutting point can be expressed as

1 $$\:{E}_{g}(x,\:y,\:z)\:=\:{E}_{pos}\:+\:{E}_{orient}\:+\:{E}_{calib}$$

where $\:{E}_{pos}$ represents positional errors, $\:{E}_{orient}$ denotes orientation errors, and $\:{E}_{calib}$ accounts for calibration deviations^19^.

Thermal errors emerge as the dominant contributor under continuous operation, accounting for 40–70% of total machining inaccuracies^20^. Heat generation during cutting induces non-uniform temperature distributions across the machine structure, producing thermal expansion and structural distortion. The thermal displacement at coordinate position (x, y, z) follows the relationship

2 $$\:\varDelta\:T(x,\:y,\:z,\:t)\:=\:\sum\:_{i\:=\:1}^{n}{\alpha\:}_{i}\:\cdot\:{L}_{i}\:\cdot\:{\varDelta\:T}_{i}\left(t\right)$$

where $\:{\alpha\:}_{i}$ denotes thermal expansion coefficients, $\:{L}_{i}$ represents structural lengths, and $\:{\varDelta\:T}_{i}$(t) indicates temperature variations over time t^21^.

Force-induced deformation errors arise from cutting forces exerted during material removal, producing elastic deflections in both workpiece and tool-holder systems. The magnitude of force-related displacement depends on system stiffness and the instantaneous cutting load. The deformation can be modelled as

3 $$\:{\delta\:}_{F}\:=\:\frac{{F}_{c}}{{k}_{sys}}\:=\:\frac{{K}_{c}\:\cdot\:{a}_{p}\:\cdot\:f}{{k}_{sys}}$$

where $\:{F}_{c}$ represents cutting force, $\:{k}_{sys}$ denotes system stiffness, $\:{K}_{c}$ is specific cutting pressure, $\:{a}_{p}$ indicates depth of cut, and f represents feed rate^22^.

Tool wear progressively degrades cutting edge geometry, introducing time-dependent errors that accumulate throughout machining cycles. Flank wear directly correlates with dimensional overshooting, while crater wear alters chip formation mechanics and force distributions. The evolution of wear-induced error follows

4 $$\:{E}_{w}\left(t\right)\:=\:{k}_{w}\:\cdot\:VB\left(t\right)\:=\:{k}_{w}\:\cdot\:{C}_{w}\:\cdot\:{v}^{\alpha\:}\:\cdot\:{f}^{\beta\:}\:\cdot\:{a}_{p}^{\gamma\:}\:\cdot\:t$$

where $\:{E}_{w}$ denotes wear-related error, VB represents flank wear land width, $\:{k}_{w}$ is the wear coefficient, $\:{C}_{w}$ encompasses material-dependent constants, v indicates cutting speed, and α, β, γ are empirical exponents^23^.

The coupling among these error sources creates synergistic effects that amplify individual contributions. Thermal expansion alters geometric accuracies, modified geometries shift force application points and thereby change deformation patterns, and accelerated tool wear under elevated temperatures further compounds dimensional deviations. The total machining error manifests as a nonlinear superposition

5 $$\:{E}_{total}\:=\:{E}_{g}\:+\:{E}_{T}\:+\:{E}_{F}\:+\:{E}_{w}\:+\:\sum\:_{i\:\ne\:\:j}^{}{\phi\:}_{ij}({E}_{i},\:{E}_{j})$$

where $\:{\phi\:}_{ij}$ represents coupling functions between error components $\:{E}_{i}$ and $\:{E}_{j}$. Capturing these intricate interactions becomes essential for effective compensation, since isolated treatment of individual error sources proves insufficient under real-world conditions where simultaneous disturbances prevail.

### Machining error prediction and modeling methods

Error prediction methodologies in CNC turning have evolved through three distinct paradigms—empirical, physics-based, and data-driven models—each carrying its own balance of strengths and limitations. Empirical models, derived from extensive experimental observations, establish simplified relationships between process parameters and resulting errors through regression analysis^24^. A typical empirical formulation takes the form

6 $$\:{E}_{pred}\:=\:{C}_{0}\:+\:{C}_{1}{v}^{{a}_{1}}\:+\:{C}_{2}{f}^{{a}_{2}}\:+\:{C}_{3}{a}_{p}^{{a}_{3}}\:+\:{C}_{4}{t}^{{a}_{4}}$$

where $\:{C}_{i}$ and $\:{a}_{i}$ represent empirically determined coefficients and exponents, respectively. Such models are computationally efficient and easy to implement, yet their applicability stays confined to the calibration window from which they were estimated^25^. Extrapolation beyond validated parameter ranges frequently yields unreliable predictions, severely limiting their utility in adaptive compensation systems.

Physics-based models, grounded in fundamental mechanics and heat-transfer principles, attempt to capture the underlying error-generation mechanisms through first-principle equations^26^. They incorporate material properties, thermal conductivity, elastic moduli, and cutting mechanics to simulate error evolution. The governing equation for thermal–mechanical coupling typically reads

7 $$\:\rho\:\:{c}_{p}\:\frac{\partial\:T}{\partial\:t}\:=\:\nabla\:\:\cdot\:(k\:\nabla\:T)\:+\:{Q}_{gen}\:-\:\sigma\:\:{\epsilon\:}_{th}$$

where ρ denotes material density, $\:{c}_{p}$ represents specific heat capacity, k indicates thermal conductivity, $\:{Q}_{gen}$ describes the heat-generation rate, and $\:{\epsilon\:}_{th}$ signifies thermal strain. Physics-based approaches provide interpretable insight into causality and stay valid across broader operational ranges, but they demand substantial computational resources and detailed knowledge of material characteristics, boundary conditions, and contact phenomena—information often unavailable or impractical to obtain in production^27^.

Data-driven models have emerged as compelling alternatives, exploiting machine-learning algorithms to extract predictive patterns directly from historical machining data without requiring explicit physical formulations^28^. Neural networks, support vector machines, and ensemble methods construct complex nonlinear mappings between input features (cutting parameters, sensor signals, machine states) and output errors. A generic data-driven predictor follows

8 $$\:\hat{E}\:=\:{f}_{ML}(x;\:\varTheta\:)\:=\:{f}_{ML}\left(\right[v,\:f,\:{a}_{p},\:T,\:{F}_{c},\:\dots\:\:];\:\varTheta\:)$$

where x represents the feature vector, Θ encompasses model parameters, and $\:{f}_{ML}$ denotes the learned mapping. These models excel at capturing intricate relationships that evade analytical description, automatically adapting to system-specific characteristics through training^29^. They face criticism, however, on interpretability and data hunger, requiring extensive labelled datasets for adequate generalisation.

Recent investigations into hybrid modelling strategies attempt to combine advantages from multiple paradigms. Physics-informed neural networks embed governing equations as constraints within learning architectures, reducing data requirements while preserving physical consistency^30^. Transfer-learning techniques enable knowledge migration from well-characterised systems to new configurations, accelerating model development cycles. Bayesian frameworks quantify prediction uncertainties, providing confidence intervals crucial for risk-sensitive compensation decisions. The expression of prediction uncertainty can be written as

9 $$\:P\left(E\:\right|\:x)\:=\:{\int\:}_{}^{}P\left(E\:\right|\:x,\:\varTheta\:\left)\:P\right(\varTheta\:\:\left|\:D\right)\:d\varTheta\:$$

where D represents the training dataset and P(Θ|D) denotes the posterior parameter distribution. Despite these advances, achieving robust real-time prediction across diverse machining scenarios remains elusive. Model degradation under tool-wear progression, inadequate handling of abrupt disturbances, and limited capacity for online adaptation continue to challenge practical deployment.

### Error compensation strategies and methods

Error compensation, at its core, counteracts predicted or measured deviations through corrective adjustments to machining commands or tool trajectories^31^. The principle can be written as

10 $$\:{P}_{compensated}\:=\:{P}_{nominal}\:-\:{E}_{predicted}$$

where $\:{P}_{nominal}$ represents the original tool path coordinates and $\:{E}_{predicted}$ denotes the anticipated error vector. Successful compensation hinges on accurate error estimation and on timely implementation of corrective actions within the control loop.

Static compensation strategies apply predetermined correction values from offline calibration procedures or lookup tables under specific operating conditions^32^. These approaches assume that error patterns remain invariant throughout machining, updating compensation only at periodic recalibration intervals. While they offer simplicity and minimal computational burden, static methods prove inadequate against time-varying disturbances such as progressive tool wear or fluctuating thermal states. Their effectiveness deteriorates rapidly as the actual conditions diverge from the calibration scenario, leading to systematic residual errors that accumulate over extended production runs.

Dynamic compensation introduces real-time error monitoring through sensor integration, enabling continuous adjustment of correction values based on measured process variables^33^. Temperature sensors, force transducers, and displacement gauges provide feedback signals that drive compensation algorithms. The dynamic correction follows

11 $$\:\varDelta\:P\left(t\right)\:=\:{K}_{p}\:e\left(t\right)\:+\:{K}_{i}\:{\int\:}_{0}^{t}e\left(\tau\:\right)\:d\tau\:\:+\:{K}_{d}\:\frac{d\:e\left(t\right)}{d\:t}$$

where e(t) represents instantaneous error and $\:{K}_{p}$, $\:{K}_{i}$, $\:{K}_{d}$ denote controller gains. This closed-loop architecture responds to process variations more effectively than static schemes, yet conventional dynamic compensators rely on fixed control parameters that may not suit varying operational regimes^34^. Tuning those parameters demands considerable expertise and remains application-specific, limiting generalisation across different workpiece materials or cutting conditions.

Adaptive compensation goes beyond fixed-parameter control by autonomously modifying compensation strategies according to evolving machining states^35^. Self-tuning regulators, model reference adaptive controllers, and gain-scheduling techniques exemplify this category. These methods adjust controller characteristics in response to identified system dynamics or performance metrics; nevertheless, traditional adaptive approaches assume parametric uncertainty within predefined model structures, which is uncomfortable when facing unmodelled dynamics or abrupt regime changes characteristic of real machining environments.

Intelligent optimisation algorithms have recently gained traction for compensation challenges that evade conventional control paradigms. Neural networks approximate complex nonlinear error mappings without requiring explicit mathematical models, while evolutionary algorithms navigate multi-modal solution spaces to identify optimal compensation parameters^36^. The optimisation objective typically appears as

12 $$\:\varTheta\:*\:=\:arg\:{min}_{\varTheta\:}\:\{\sum\:_{i\:=\:1}^{N}{\left|{E}_{i}\right(\varTheta\:\left)\right|}^{2}\:+\:\lambda\:\:R(\varTheta\:\left)\right\}$$

where Θ encompasses compensation parameters, $\:{E}_{i}$ denotes residual errors, and R(Θ) represents regularisation terms. These intelligent techniques show real strength in handling high-dimensional parameter spaces and capturing intricate relationships between process variables and compensation efficacy. Their learning capacity supports continuous improvement through accumulated experience. Despite encouraging results in controlled experiments, industrial adoption remains limited due to concerns over training stability, convergence guarantees, and interpretability of learned behaviours—the very gaps that motivate hybrid frameworks combining complementary strengths.

## Error compensation model based on deep reinforcement learning and genetic algorithm fusion

### Deep reinforcement learning compensation model design

**Fig. 1:**
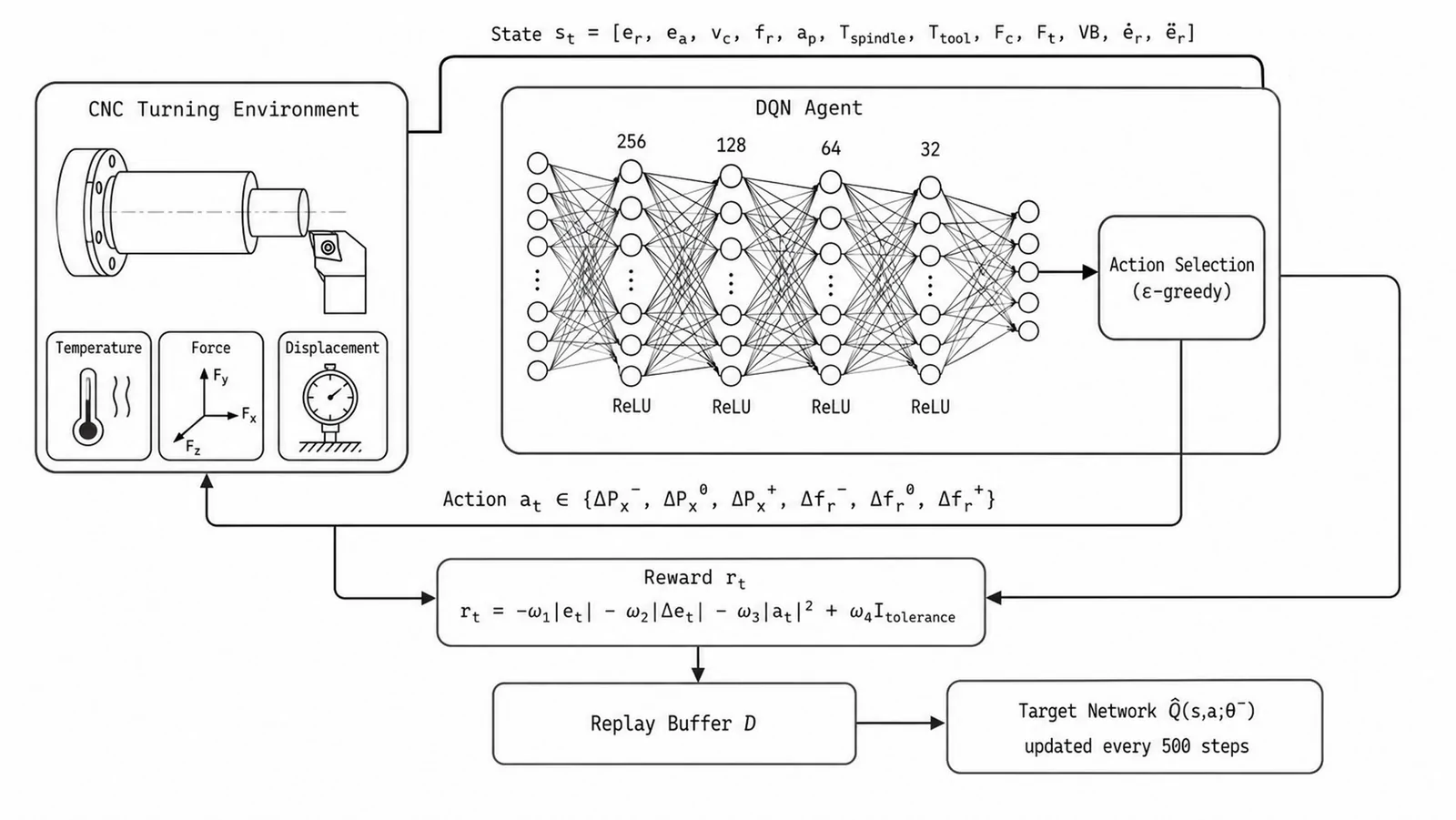
Deep reinforcement learning compensation framework architecture, showing state inputs, action outputs, and reward propagation.

We establish a deep Q-network (DQN) framework tailored for machining error compensation, in which the agent learns optimal adjustment policies through iterative interaction with the CNC turning environment^37^. Figure 1 illustrates the overall architecture of the proposed DRL-based compensation system, depicting the information flow between the machining environment, the neural network, and the decision-making components, with explicit annotation of state, action, and reward channels.

The state space encapsulates comprehensive machining-condition descriptors that influence error formation. At time step t, the state vector $\:{s}_{t}$ incorporates

13 $$\:{s}_{t}\:=\:{\left[{e}_{r}\right(t),\:{e}_{a}(t),\:{v}_{c},\:{f}_{r},\:{a}_{p},\:{T}_{spindle},\:{T}_{tool},\:{F}_{c},\:{F}_{t},\:VB,\:\dot{e}_{r},\:\ddot{e}_{r}]}^{T}$$

where $\:{e}_{r}$ and $\:{e}_{a}$ represent radial and axial dimensional errors, $\:{v}_{c}$ denotes cutting velocity, $\:{f}_{r}$ indicates feed rate, $\:{a}_{p}$ signifies depth of cut, $\:{T}_{spindle}$ and $\:{T}_{tool}$ capture thermal states, $\:{F}_{c}$ and $\:{F}_{t}$ reflect cutting and thrust forces, VB quantifies tool wear, while $\:\dot{e}_{r}$ and $\:\ddot{e}_{r}$ provide error rate and acceleration information^38^. This multi-dimensional representation enables the agent to perceive both instantaneous conditions and dynamic trends essential for proactive compensation.

The action space defines feasible compensation adjustments the agent can execute. To balance exploration breadth against computational tractability, we discretise continuous compensation into a finite set of incremental modifications

14 $$\:{a}_{t}\:\in\:\:A\:=\:\{\:{\varDelta\:P}_{x}^{-},\:{\varDelta\:P}_{x}^{0},\:{\varDelta\:P}_{x}^{+},\:{\varDelta\:f}_{r}^{-},\:{\varDelta\:f}_{r}^{0},\:{\varDelta\:f}_{r}^{+}\:\}$$

where $\:{\varDelta\:P}_{x}$ denotes radial position adjustment and $\:{\varDelta\:f}_{r}$ represents feed rate modification, with superscripts indicating decrease, maintain, or increase actions respectively.

The reward function quantifies compensation effectiveness, guiding learning toward error minimisation while penalising excessive adjustments that might destabilise machining. We define it as

15 $$\:{r}_{t}\:=\:-\:{\omega\:}_{1}\:\left|{e}_{t}\right|\:-\:{\omega\:}_{2}\:\left|\varDelta\:{e}_{t}\right|\:-\:{\omega\:}_{3}{\:\left|{a}_{t}\right|}^{2}\:+\:{\omega\:}_{4}\:{I}_{tolerance}\left({e}_{t}\right)$$

where $\:{\omega\:}_{i}$ are weighting coefficients, $\:{\varDelta\:e}_{t}\:=\:{e}_{t}\:-\:{e}_{t\:-\:1}$ captures error change, and $\:{I}_{tolerance}$ provides bonus rewards when errors fall within tolerance bands^39^. This multi-objective formulation encourages both accuracy and stability.

Our neural-network architecture comprises four fully-connected hidden layers with 256, 128, 64, and 32 neurons respectively, employing rectified linear unit (ReLU) activations. The network maps states to action-value estimates through

16 $$\:Q({s}_{t},\:a;\:\theta\:)\:=\:{W}_{4}\:\cdot\:ReLU({W}_{3}\:\cdot\:ReLU({W}_{2}\:\cdot\:ReLU({W}_{1}\:\cdot\:{s}_{t})\left)\right)$$

where θ = {$\:{W}_{1}$, $\:{W}_{2}$, $\:{W}_{3}$, $\:{W}_{4}$} represents network parameters trained to approximate optimal action values. Table 1 summarises the hyperparameter configuration. Detailed engineering rationale for each value is moved to the Appendix; here we only emphasise that learning-rate α and reward-weight $\:{\omega\:}_{1}$ dominate behaviour, with the remaining parameters chosen by a two-stage TPE-then-grid procedure described in Sect.  3.2.

**Table 1:** Deep reinforcement learning model parameter configuration.

Parameter	Value	Description
Learning rate α	0.0001	Gradient descent step size
Discount factor γ	0.95	Future reward weighting
Replay buffer size	50,000	Experience storage capacity
Batch size	64	Mini-batch sampling size
Target network update frequency	500 steps	Synchronisation interval
Exploration rate ε_init	1.0	Initial exploration probability
Exploration decay rate	0.995	Exponential decay coefficient
Minimum exploration rate ε_min	0.05	Lower bound for exploration

Experience replay mitigates temporal correlation issues inherent in sequential machining data by storing transitions ($\:{s}_{t},\:{a}_{t},\:{r}_{t},\:{s}_{t\:+\:1}$) in a replay buffer D and randomly sampling mini-batches during training^40^. The loss function for network optimisation becomes

17 $$\:L\left(\theta\:\right)\:=\:{E}_{(s,\:a,\:r,\:s{\prime\:})\:\sim\:\:D}\:\left[{(r\:+\:\gamma\:\:{max}_{a{\prime\:}}\:Q(s{\prime\:},\:a{\prime\:};\:{\theta\:}^{-})\:-\:Q(s,\:a;\:\theta\:\left)\right)}^{2}\right]$$

where $\:{\theta\:}^{-}$ denotes target-network parameters updated periodically to stabilise training dynamics^41^. This decoupling between evaluation and target networks prevents the divergence that would otherwise plague value-function approximation. Through iterative policy improvement the DRL agent autonomously discovers compensation strategies that adapt to evolving machining states, refining its decision-making as operational experience accumulates.

### Genetic algorithm optimization strategy

**Fig. 2:**
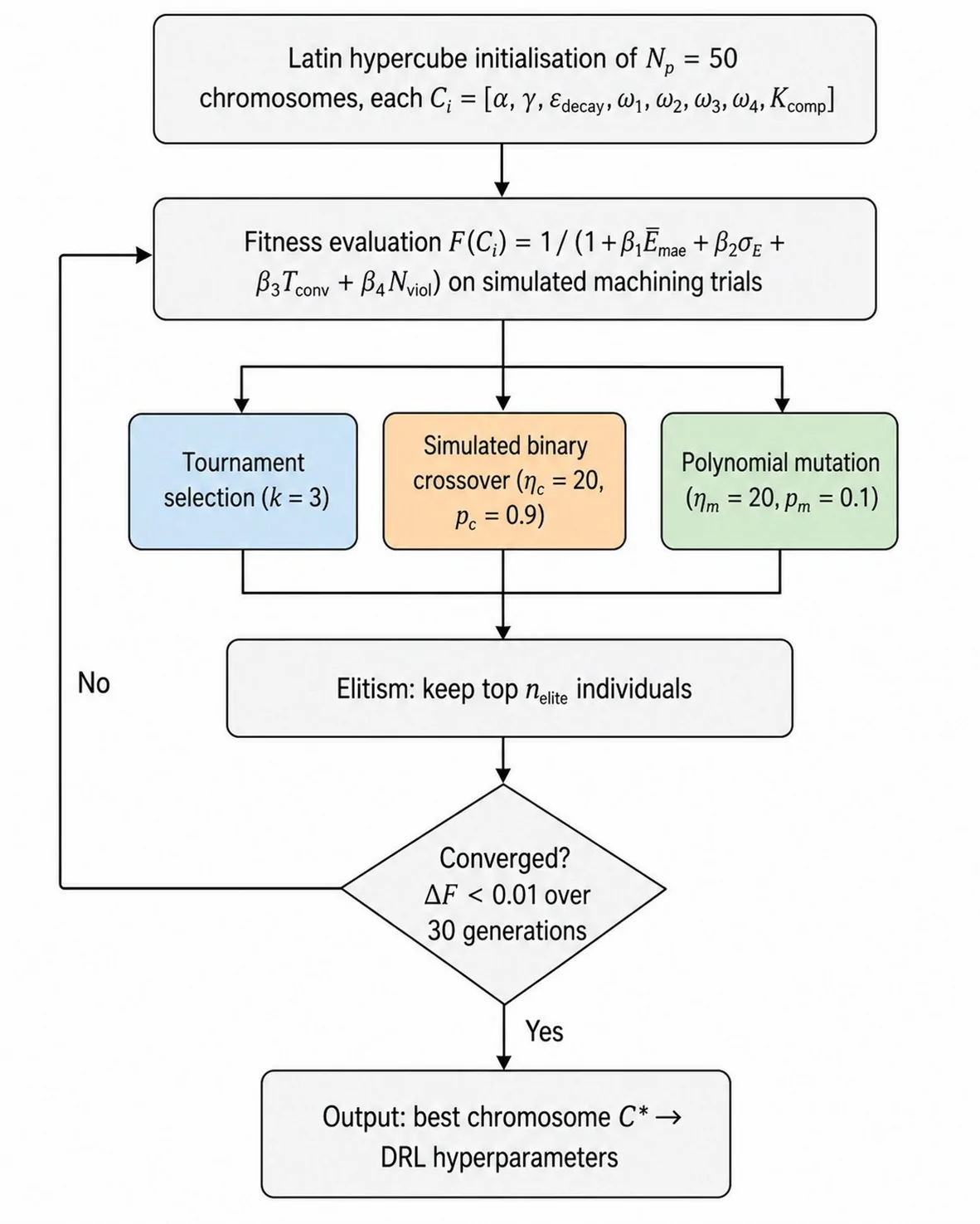
Genetic algorithm optimisation workflow with selection, crossover, mutation, elitism, and fitness coupling back to DRL.

Genetic algorithms serve as global optimisation engines within our hybrid framework, addressing the hyperparameter selection and compensation-gain tuning that critically influence DRL performance^42^. Figure 2 presents the complete evolutionary optimisation workflow, with selection, crossover, mutation, and elitism preservation explicitly shown alongside the fitness evaluation channel that ties the GA back to the DRL agent.

Through this evolution loop, the algorithm explores the parameter search space while exploiting promising regions identified in previous generations.

We adopt real-valued encoding to represent candidate solutions, where each chromosome $\:{C}_{i}$ carries both DRL hyperparameters and compensation gains. The chromosome is defined as

18 $$\:{C}_{i}\:=\:[\alpha\:,\:\gamma\:,\:{\epsilon\:}_{decay},\:{\omega\:}_{1},\:{\omega\:}_{2},\:{\omega\:}_{3},\:{\omega\:}_{4},\:{K}_{comp}]$$

where the genes have the following meaning: α is the DRL learning rate, γ is the discount factor governing the weight given to future rewards, $\:{\epsilon\:}_{decay}$ is the exploration-rate decay coefficient that schedules the shift from exploration to exploitation, $\:{\omega\:}_{1}$ through $\:{\omega\:}_{4}$ are the four reward-function weights introduced in Eq. (15) (penalising error magnitude, error change, control effort, and rewarding in-tolerance operation, respectively), and $\:{K}_{comp}$ is a scalar gain applied to the action output before it reaches the CNC controller. Real-valued encoding eliminates the discretisation artefacts that binary representations would introduce^43^, and each gene is normalised so that evolutionary pressure remains comparable across parameters with disparate numerical scales.

The fitness function evaluates chromosome quality through comprehensive performance metrics, obtained by deploying corresponding parameter configurations in simulated machining trials

19 $$\:F\left({C}_{i}\right)\:=\:\frac{1}{1\:+\:{\beta\:}_{1}\:\bar{E}_{mae}\:+\:{\beta\:}_{2}\:{\sigma\:}_{E}\:+\:{\beta\:}_{3}\:{T}_{conv}\:+\:{\beta\:}_{4}\:{N}_{viol}}$$

where $\:\bar{E}_{mae}$ denotes mean absolute error across test episodes, $\:{\sigma\:}_{E}$ represents the error standard deviation, $\:{T}_{conv}$ measures convergence time, $\:{N}_{viol}$ counts tolerance violations, and $\:{\beta\:}_{j}$ are weighting coefficients prioritising accuracy over speed. This multi-criterion fitness rewards solutions that balance precision, robustness, and learning efficiency simultaneously.

Tournament selection determines parent chromosomes for reproduction, with k individuals competing and the fittest advancing^44^. This maintains selection pressure while preserving population diversity better than rank-based alternatives

20 $$\:{P}_{select}\left(i\right)\:=\:\frac{1}{{N}_{p}}\:\cdot\:{\left(\frac{F\left({C}_{i}\right)}{\sum\:_{j\:\in\:\:Tournament}^{}F\left({C}_{j}\right)}\right)}^{\tau\:}$$

where $\:{N}_{p}$ denotes population size and τ controls selection intensity, so that higher fitness values yield proportionally greater selection probabilities.

Simulated binary crossover (SBX) generates offspring by blending parent genes according to polynomial probability distributions that respect search-space boundaries

21 $$\:{C}_{offspring}\:=\:0.5\:\left[\right(1\:+\:{\beta\:}_{q})\:{C}_{p1}\:+\:(1\:-\:{\beta\:}_{q}\left)\:{C}_{p2}\right]$$

where $\:{C}_{p1}$ and $\:{C}_{p2}$ represent parent chromosomes and $\:{\beta\:}_{q}$ follows a distribution determined by the crossover parameter $\:{\eta\:}_{c}$, enabling controlled exploration around parental solutions. Table 2 lists the specific parameter settings governing genetic operations; rationale for the values is again deferred to the Appendix.

**Table 2:** Genetic algorithm parameter configuration.

Parameter	Value	Description
Population size N_p	50	Number of candidate solutions
Tournament size k	3	Competitors in selection
Crossover probability p_c	0.9	Recombination likelihood
Mutation probability p_m	0.1	Gene alteration rate
Crossover distribution index η_c	20	SBX spread control
Mutation distribution index η_m	20	Polynomial mutation spread

Polynomial mutation introduces stochastic perturbations to individual genes, enabling escape from local optima while respecting parameter bounds

22 $$\:{C}_{i,\:mutated}\:=\:{C}_{i}\:+\:{\delta\:}_{i}\:\cdot\:({C}_{i,\:max}\:-\:{C}_{i,\:min})$$

where $\:{\delta\:}_{i}$ follows a polynomial distribution controlled by $\:{\eta\:}_{m}$, producing small changes with high probability and large jumps occasionally^45^. Population initialisation employs Latin hypercube sampling to ensure comprehensive coverage of the parameter space from generation zero, avoiding clustering that random initialisation might produce. Elitism preservation directly transfers the top $\:{n}_{elite}$ individuals to the next generation unchanged, safeguarding the best discovered solutions from degradation through genetic operations.

For reproducibility, the values populating Tables 1 and 2 were not chosen heuristically. We first ran a sparse Bayesian search using a Tree-structured Parzen Estimator across 60 trials over a wide search space, with validation MAE on the 15% held-out set as surrogate objective. Promising regions identified by the TPE were then refined through 5-fold cross-validation grid search across narrower intervals. The sensitivity analysis around the chosen values, reported in Table 3, indicates that learning rate α and the first reward weight $\:{\omega\:}_{1}$ dominate convergence behaviour, while crossover index $\:{\eta\:}_{c}$ and tournament size k contribute only marginally within ± 20% perturbations. To check that these settings are not seed-specific, we repeated the calibration on five distinct random seeds and observed that the resulting hyperparameters varied by less than 8% across seeds.

**Table 3:** Hyperparameter sensitivity analysis around calibrated values.

Hyperparameter	Calibrated value	ΔMAE at ± 20% (µm)	Sensitivity rank
Learning rate α	0.0001	+ 1.42 / +1.78	1 (high)
Reward weight ω₁	1.00	+ 1.05 / +1.31	2 (high)
Discount factor γ	0.95	+ 0.61 / +0.79	3 (medium)
Population size N_p	50	+ 0.34 / +0.42	4 (medium)
Crossover index η_c	20	+ 0.18 / +0.22	5 (low)
Tournament size k	3	+ 0.13 / +0.19	6 (low)

As Table 3 shows, the learning rate and the dominant reward weight account for most of the performance variance, suggesting that practitioners adapting our framework to a new platform should focus calibration effort on these two parameters first.

### Fusion algorithm implementation and compensation workflow

The proposed DRL-GA fusion framework orchestrates bidirectional cooperation between reinforcement learning and evolutionary optimisation, creating a system in which each methodology compensates for the other’s weaknesses^46^. Our integration strategy operates on dual timescales: DRL executes rapid online adaptation responding to immediate machining variations, while GA conducts periodic offline optimisation refining the underlying policy structure and hyperparameters. This hierarchical architecture balances real-time responsiveness with systematic performance enhancement.

**Fig. 3:**
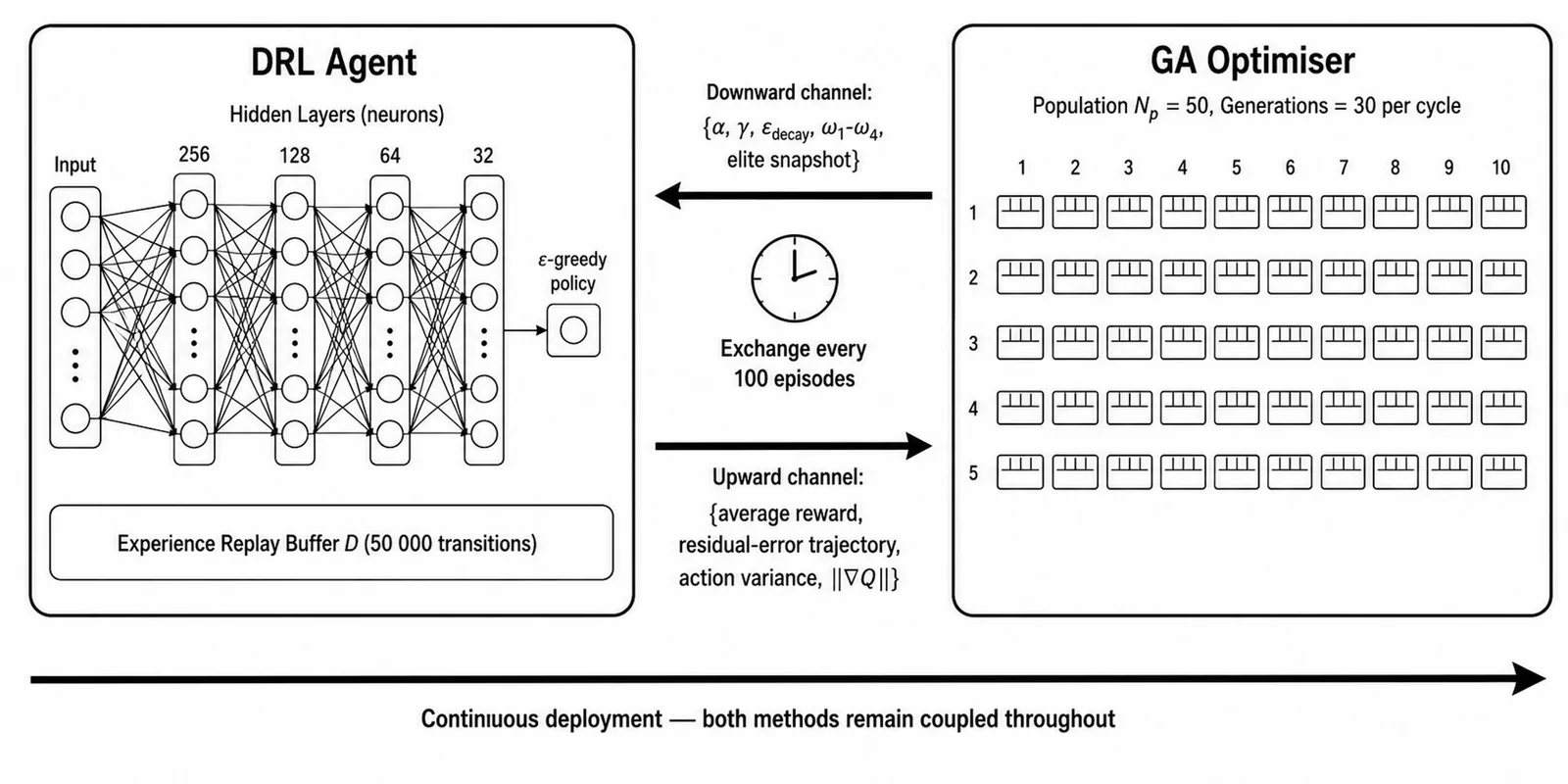
Bidirectional information exchange between the DRL agent (four hidden layers of 256-128-64-32 neurons) and the GA optimiser (50 individuals over 30 generations), annotated with exchanged variables.

Two information channels operate concurrently to realise this bidirectional cooperation, and the distinction between them deserves to be made explicit. The downward channel, running from GA to DRL, transmits three quantities at every offline cycle: an updated hyperparameter vector (learning rate, discount factor, exploration decay), refreshed reward weights $\:{\omega\:}_{1}$ through $\:{\omega\:}_{4}$, and an elite-individual snapshot that serves as a soft anchor for policy regularisation. The upward channel, from DRL to GA, returns four signals harvested over the most recent operating window: average reward, residual error trajectory, action-variance statistics, and the gradient norm of the Q-network. These four signals enter GA’s fitness evaluation through Eq. (19) as empirical priors, biasing chromosome selection toward configurations whose predicted behaviour matches what was actually observed online. The exchange happens once per evaluation window of 100 episodes, yet its influence is continuous because the DRL agent keeps learning between two GA cycles using the most recently delivered hyperparameters. Figure 3 visualises this bidirectional coupling, with layer counts, generation counts, and exchanged variables annotated directly on the diagram so that readers can trace the data flow without consulting the surrounding text. This is what differentiates our scheme from sequential pipelines in which GA runs once and is then discarded—here the two methods remain coupled throughout deployment.

The initialisation phase begins by launching the genetic algorithm to establish a competent starting configuration for the DRL agent. Rather than arbitrarily selecting hyperparameters, GA explores the configuration space through 50 generations of evolution, identifying parameter sets that demonstrate promising convergence characteristics in preliminary simulation trials. The best-performing chromosome from this initial run seeds the DRL network parameters

23 $$\:[\alpha\:*,\:\gamma\:*,\:{\epsilon\:}_{decay}*,\:\omega\:*,\:{K}_{comp}*]\:=\:arg\:{max}_{C\:\in\:\:{Population}_{final}}\:F\left(C\right)$$

This informed initialisation substantially reduces the exploration burden during online learning, letting the DRL agent begin deployment with parameters already tuned toward effective compensation^47^.

Online learning proceeds through continuous machining episodes where the DRL agent interacts with the physical CNC system. At each control cycle, the agent observes state $\:{s}_{t}$, selects compensation action $\:{a}_{t}$ via the ε-greedy policy, receives reward $\:{r}_{t}$, and transitions to $\:{s}_{t\:+\:1}$. Experience tuples accumulate in the replay buffer, and mini-batch gradient descent updates the Q-network according to Eq. (17). The adaptive exploration rate decays following

24 $$\:{\epsilon\:}_{t}\:=\:max({\epsilon\:}_{min},\:{\epsilon\:}_{init}\:\cdot\:{\rho\:}^{\frac{t}{{\tau\:}_{decay}}})$$

where ρ denotes decay rate and $\:{\tau\:}_{decay}$ controls temporal scaling, gradually shifting from exploration toward exploitation as the agent gains experience.

Offline optimisation activates periodically when performance metrics indicate potential for improvement or when accumulated experience reaches predefined thresholds. The GA receives performance statistics from recent DRL operation—average error, convergence speed, and policy stability—and evolves a new population seeking enhanced configurations. Critically, the current DRL parameters enter the GA population as an elite individual, ensuring optimisation cannot degrade existing performance. After evolutionary iterations converge, the GA returns an improved parameter set that updates the DRL agent through

25 $$\:{\theta\:}_{DRL}\:\leftarrow\:\:{\theta\:}_{DRL}\:+\:{\eta\:}_{transfer}\:({\theta\:}_{GA}^{*}\:-\:{\theta\:}_{DRL})$$

where $\:{\eta\:}_{transfer}$ controls the magnitude of parameter adjustment, allowing gradual transitions that maintain operational stability^48^. Table 4 details the complete execution workflow with specific operational parameters governing each phase.

**Table 4:** Fusion algorithm execution workflow parameters.

Phase	Step	Operation	Parameter	Value
Initialisation	1	GA pretraining	Initial generations	50
Initialisation	2	Parameter transfer	Transfer rate η_init	1.0
Online Learning	3	Episode execution	Episode length	500 steps
Online Learning	4	Experience collection	Buffer update rate	Real-time
Online Learning	5	Network training	Training frequency	Every 4 steps
Offline Optimisation	6	Performance evaluation	Evaluation window	100 episodes
Offline Optimisation	7	GA activation	Trigger threshold	ΔF < 0.01
Offline Optimisation	8	Evolution execution	Optimisation generations	30
Offline Optimisation	9	Parameter update	Transfer rate η_transfer	0.3
Convergence Check	10	Termination criterion	Improvement threshold	ΔE < 0.5 μm

Convergence assessment monitors multiple indicators to determine when the fusion algorithm achieves satisfactory compensation. The primary criterion examines error-reduction stagnation over consecutive evaluation windows, given by

26 $$\:Converged\:\iff\:\:\frac{\left|\bar{E}_{window}\right(t)\:-\:\bar{E}_{window}(t\:-\:1\left)\right|}{\bar{E}_{window}(t\:-\:1)}\:<\:{\xi\:}_{conv},\:\:\forall\:t\:\in\:\:[{t}_{check},\:{t}_{check}\:+\:\varDelta\:{t}_{verify}]$$

where $\:{\xi\:}_{conv}$ = 0.02 represents the relative improvement threshold and $\:\varDelta\:{t}_{verify}$ specifies the verification duration. Secondary criteria examine policy stability through action variance and Q-value fluctuations, ensuring convergence reflects genuine capability rather than transient luck.

The complete closed-loop compensation system integrates sensor feedback, fusion-algorithm decision-making, and CNC command execution into a cohesive control architecture. Dimensional measurements from in-process gauges feed state observations, the trained DRL-GA agent computes optimal compensation adjustments, and modified G-codes execute corrective tool-path alterations. The integrated system continuously monitors machining outcomes, learns from deviations, periodically refines its optimisation strategy through genetic evolution, and adapts compensation policies to maintain dimensional accuracy despite tool-wear progression, thermal drift, and other disturbances. The fusion approach thus realises genuine adaptive intelligence exceeding what either DRL or GA could accomplish independently.

## Experimental validation and results analysis

### Experimental platform construction and data acquisition

Experimental validation was carried out on a high-precision CNC turning centre (DMG MORI CTX beta 800 TC) equipped with a FANUC 0i-TF Plus controller, providing positional resolution of 0.1 μm and repeatability accuracy within ± 2 μm^49^. The machine’s thermal stability control and rigid structural design minimise external disturbances, providing conditions suitable for isolating compensation algorithm effects from equipment-induced variability.

**Fig. 4:**
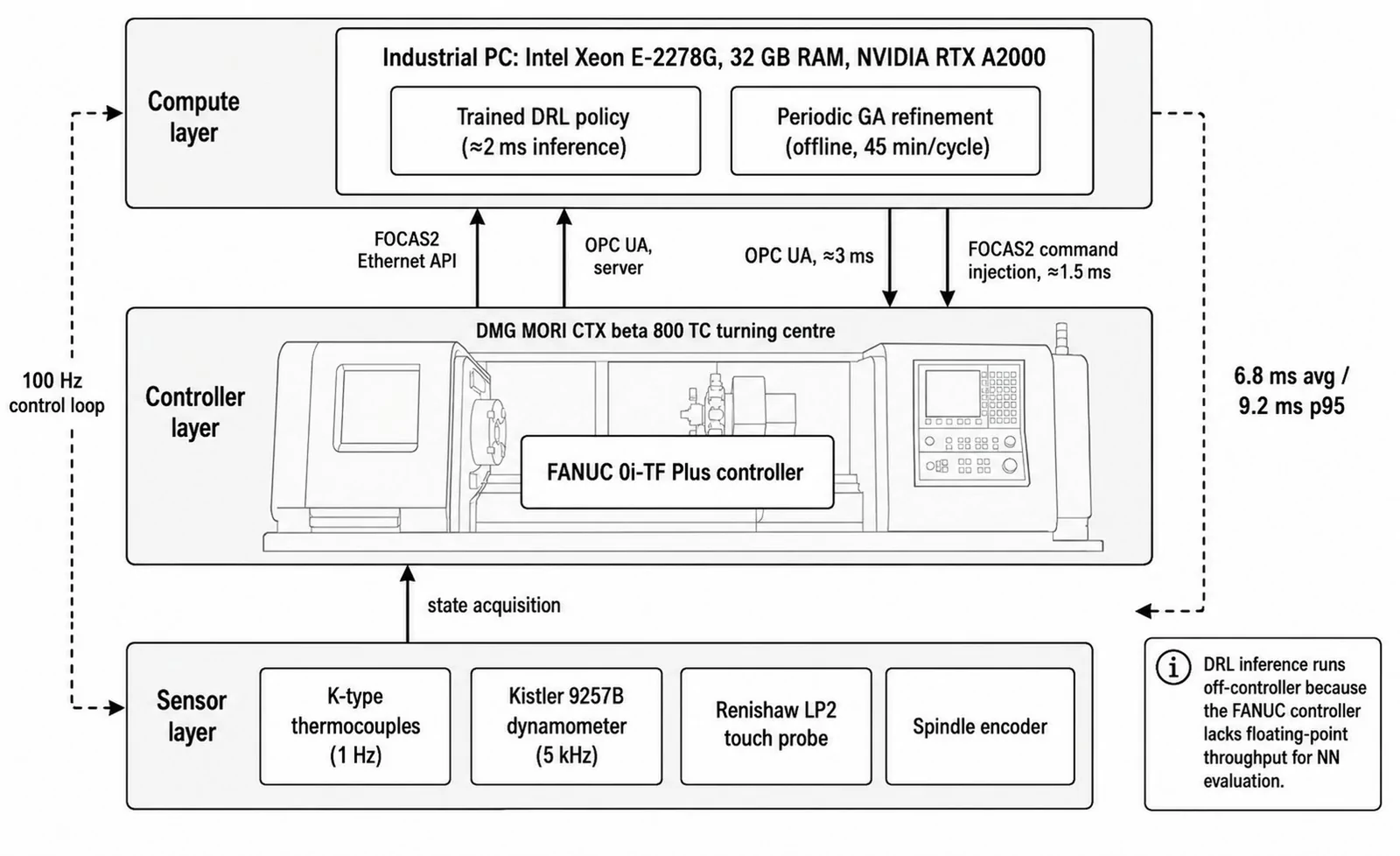
Runtime hardware-software architecture of the DRL-GA compensation system: sensors, FANUC 0i-TF Plus controller, OPC UA / FOCAS2 link, and external industrial PC running policy inference.

A clarification on the runtime architecture is in order. DRL inference does not run on the FANUC 0i-TF Plus controller itself, which lacks the floating-point throughput needed for neural-network evaluation. We deployed the trained policy on an external industrial PC (Intel Xeon E-2278G, 32 GB RAM, NVIDIA RTX A2000) connected to the controller through FANUC’s FOCAS2 Ethernet API for command injection and an OPC UA client for state acquisition. Round-trip latency, measured by hardware timestamps over 10,000 cycles, averaged 6.8 ms with a 95th-percentile of 9.2 ms—comfortably below the 10 ms budget needed to honour the 100 Hz control loop. Each cycle therefore consists of three phases: state acquisition from sensors to the industrial PC, policy evaluation on the GPU (around 2 ms), and command write-back to the controller. Figure 4 maps the hardware-software architecture in full; on-controller deployment would require either custom firmware or a dedicated FANUC AI option not available to us at the time of writing.

**Fig. 5:**
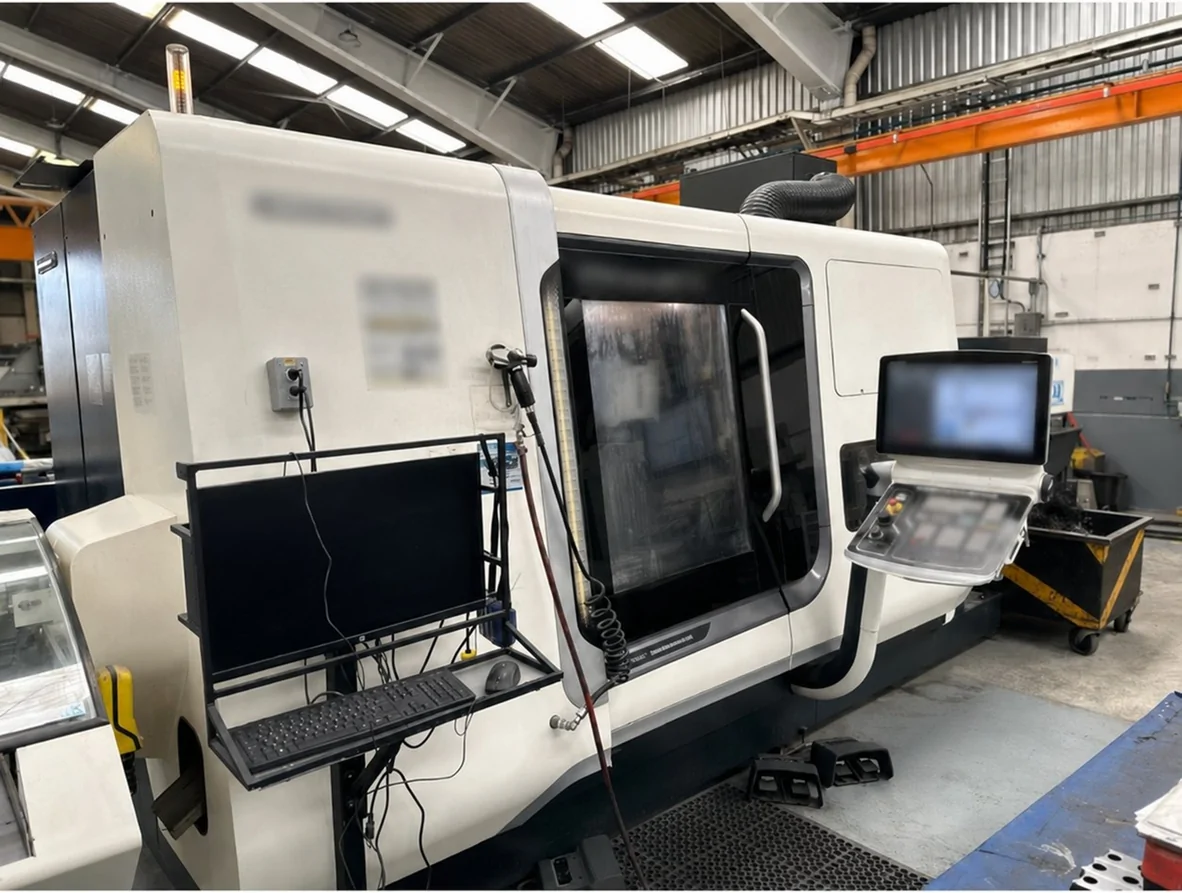
Experimental platform: DMG MORI CTX beta 800 TC turning centre with FANUC controller, operator interface, and adjacent industrial PC station.

Figure 5 shows the physical experimental platform on which all subsequent trials were conducted. The Renishaw LP2 touch probe (measurement uncertainty ± 1 μm) was mounted on the turret for in-process gauging^50^; K-type thermocouples sampled the spindle housing and tool holder at 1 Hz; the Kistler 9257B three-component dynamometer recorded cutting forces at 5 kHz; and the Mitutoyo Crysta-Apex V coordinate measuring machine (volumetric accuracy (1.7 + L/300) µm) was used for offline verification. Combining these sensing modalities provides the multi-channel state observation that the DRL-GA fusion requires.

Test workpieces consisted of cylindrical bars machined from Ti-6Al-4 V titanium alloy, selected for its industrial relevance and challenging machinability characteristics that accentuate compensation algorithm effectiveness. Specimens measured 150 mm in length and 50 mm in diameter, featuring multiple turning regions with varying depths to exercise the compensation system under diverse cutting conditions. Cemented carbide inserts (Sandvik CNMG 120408-PM 4325) with TiAlN coating served as cutting tools, replaced after every 30 machining cycles to maintain consistent wear states during training phases while allowing progressive wear evaluation during testing.

Table 5 documents the experimental conditions governing all validation trials, encompassing machine specifications, measurement equipment, workpiece properties, and cutting parameter ranges.

**Table 5:** Experimental conditions and parameter settings.

Category	Parameter	Specification
Machine Tool	Model	DMG MORI CTX beta 800 TC
Machine Tool	Controller	FANUC 0i-TF Plus
Machine Tool	Positioning accuracy	± 2 μm
Measurement	On-machine probe	Renishaw LP2 (± 1 μm)
Measurement	CMM	Mitutoyo Crysta-Apex V
Measurement	Force sensor	Kistler 9257B dynamometer
Workpiece	Material	Ti-6Al-4 V titanium alloy
Workpiece	Dimensions	Φ50 × 150 mm
Cutting Tool	Insert type	CNMG 120,408-PM 4325
Cutting Parameters	Cutting speed range	60–120 m/min
Cutting Parameters	Feed rate range	0.08–0.20 mm/rev
Cutting Parameters	Depth of cut range	0.5–2.0 mm

The experimental campaign followed a Taguchi L25(5³) orthogonal array as its backbone, augmented by a central composite design (CCD) at five interior points to recover quadratic interaction terms. Cutting speed was discretised into five levels (60, 75, 90, 105, 120 m/min), feed rate into five levels (0.08, 0.11, 0.14, 0.17, 0.20 mm/rev), and depth of cut into five levels (0.5, 0.875, 1.25, 1.625, 2.0 mm); the chosen ranges were anchored on Sandvik’s published recommendations for Ti-6Al-4 V finish turning^21^ rather than picked arbitrarily. Each parameter combination was repeated 20 times to enable variance estimation, producing 500 specimens in total. Each workpiece underwent turning operations producing stepped cylindrical features with six measurement zones located at 25 mm intervals along the length. Dimensional measurements captured radial errors at these positions immediately following machining while workpieces remained at cutting temperature, supplemented by CMM verification after thermal equilibration. This measurement strategy yields 3000 data points per experimental campaign, providing statistical robustness for performance evaluation.

Data preprocessing applied several transformations to raw measurements before algorithm training. We normalised all sensor signals to zero mean and unit variance, facilitating neural-network convergence and preventing dominance by parameters with larger numerical magnitudes. Outlier detection via interquartile-range filtering removed anomalous measurements caused by probe misalignment or transient disturbances, affecting less than 0.5% of collected data. The preprocessed dataset underwent 70-15-15 partitioning—70% for training the DRL-GA algorithm, 15% for validation during hyperparameter tuning, and 15% for final testing under previously unseen conditions. This partitioning ensures rigorous assessment of generalisation capability while providing sufficient training samples for convergence.

### Algorithm performance comparison experiments

**Fig. 6:**
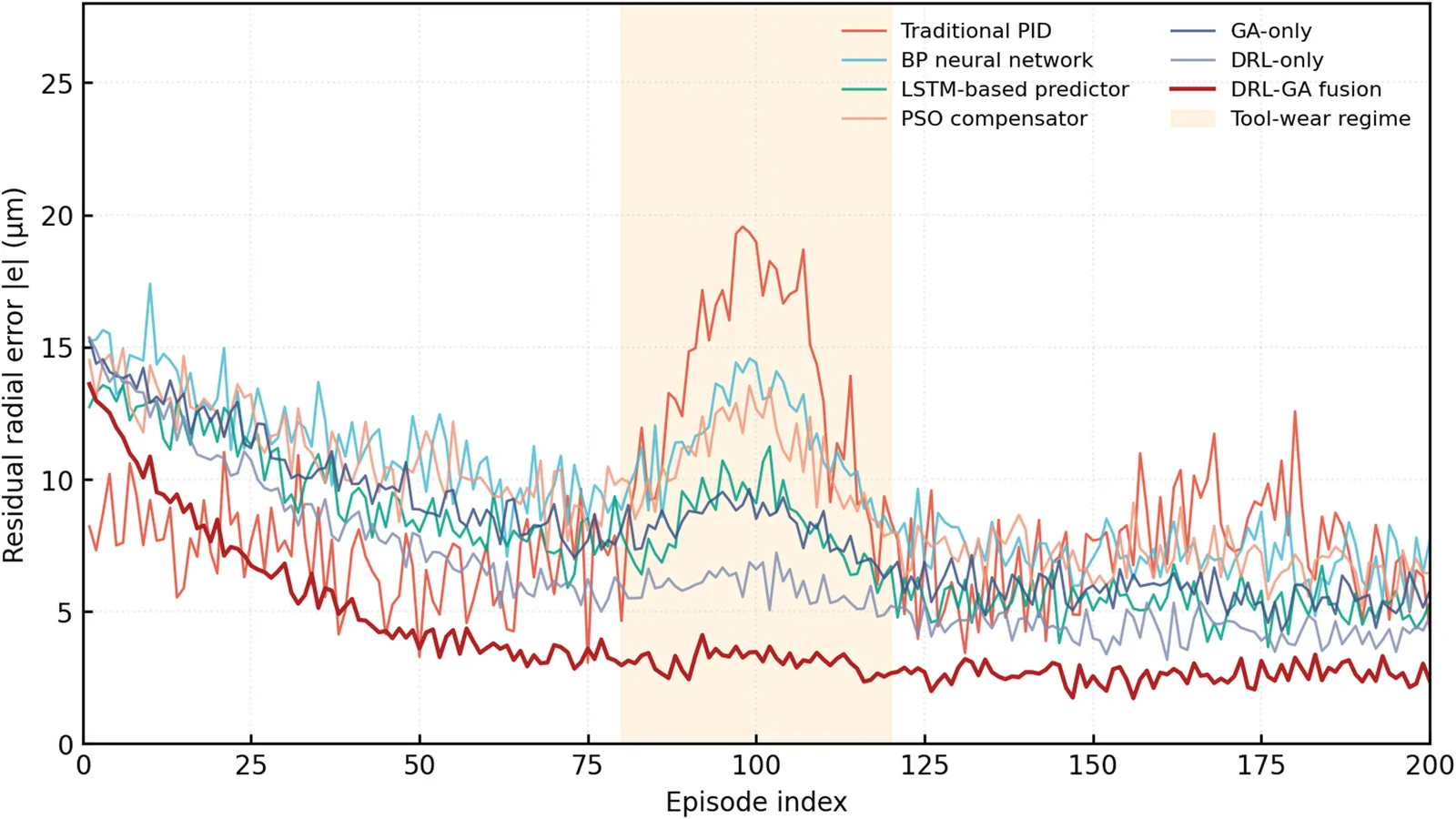
Residual radial error across 200 machining episodes for traditional PID, three mainstream baselines (BP neural network, LSTM, PSO), the two ablation configurations (GA-only, DRL-only), and the proposed DRL-GA fusion.

Comparative evaluations of the proposed DRL-GA fusion algorithm were carried out under two complementary headings. First, we benchmarked against three mainstream predictive-compensation baselines drawn from the recent literature on machining accuracy prediction: a BP neural network compensator representative of the classical shallow-network family^28,29^, an LSTM-based predictor representative of sequence-aware deep models^6^, and a particle-swarm-optimisation (PSO) compensator representative of population-based search outside the GA family^36,51^. Second, we ran ablation studies in which the DRL component or the GA component was disabled in turn, treating these as stripped-down versions of our own method rather than independently engineered systems. Each baseline was trained on the same dataset, with hyperparameters tuned through the same TPE-then-grid procedure described in Sect. "Genetic algorithm optimization strategy", and evaluated over identical cutting conditions detailed in Sect. "Experimental platform construction and data acquisition". Figure 6 presents the compensation accuracy achieved across 200 machining episodes; the shaded band marks episodes 80–120 where tool wear accelerates and the differences between methods become most pronounced.

The fusion algorithm consistently maintains the lowest dimensional errors throughout the campaign and shows the smallest dip during the tool-wear regime. The traditional PID controller exhibits the largest fluctuations, particularly during episodes 80–120, reflecting its inability to adapt compensation gains to changing system dynamics. Among the predictive baselines, the LSTM model performs best because it exploits temporal correlations in the error signal, but it still cannot match the closed-loop responsiveness of the DRL-GA fusion.

**Fig. 7:**
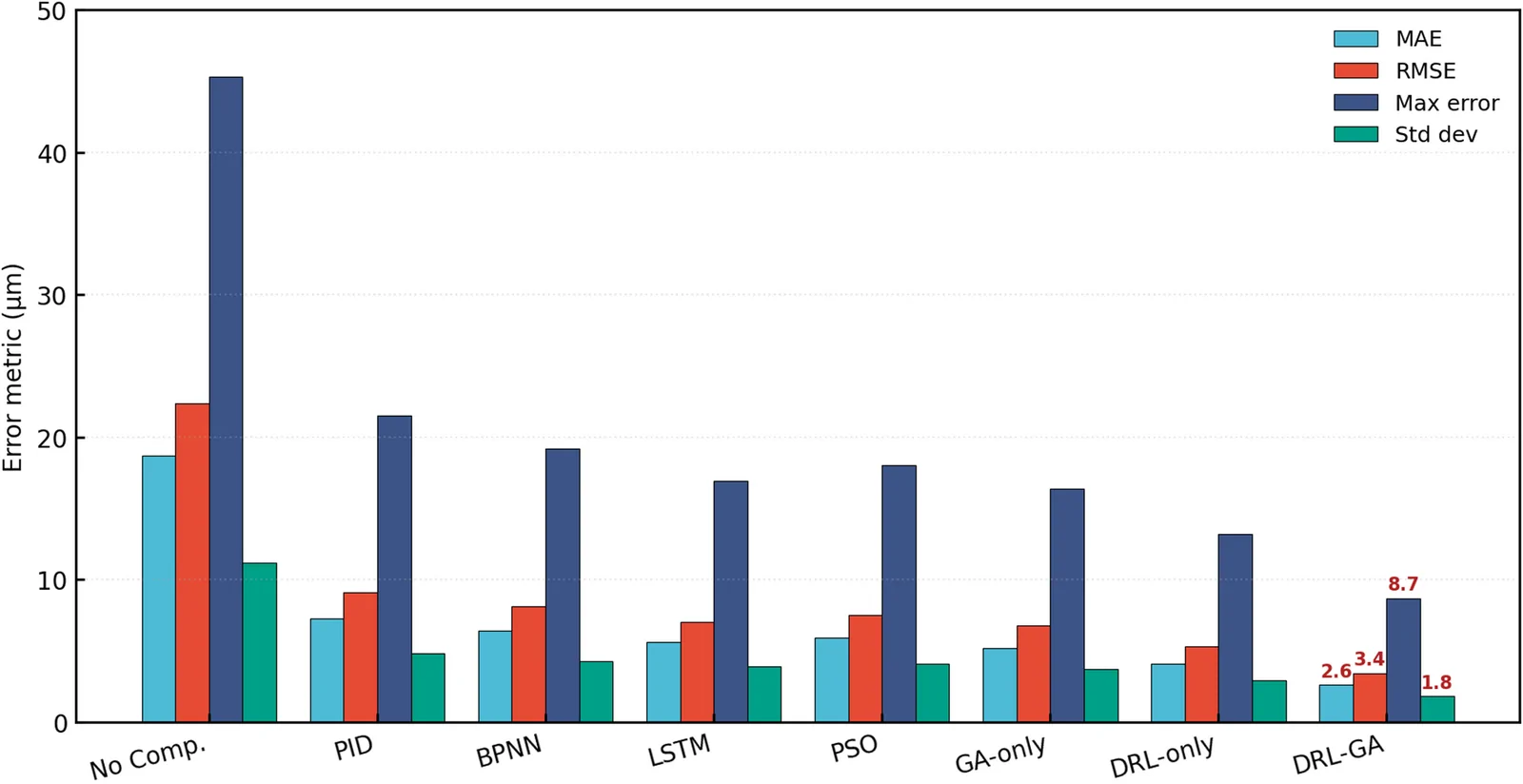
Method comparison across MAE, RMSE, maximum error, and standard deviation; DRL-GA fusion values annotated.

Quantitative performance metrics across all methods are summarised graphically in Fig. 7, where the bar chart makes the relative gaps directly visible across MAE, RMSE, maximum error, and standard deviation.

The fusion approach achieves a 64% reduction in MAE compared to traditional PID compensation and a 37% improvement over standalone DRL, with the gap to LSTM (the best non-DRL baseline) being 54%. The uncompensated baseline error of 18.7 μm underscores the necessity of active compensation for precision machining. The standard deviation of the fusion algorithm (1.8 μm) is also roughly half that of the LSTM baseline, indicating that the bidirectional coupling not only lowers average error but also tightens its distribution.

The mean absolute error and root mean square error metrics—defined respectively as the average of the absolute residuals and the square root of the mean squared residuals between uncompensated and compensated measurements—provide complementary views on average performance and sensitivity to outliers, following standard definitions consistent with ISO surface-quality terminology^52^. Our fusion algorithm achieved MAE of 2.6 μm and RMSE of 3.4 μm, demonstrating superior consistency in maintaining tight tolerances across diverse machining conditions—a property particularly valuable for high-reliability applications where occasional large errors carry disproportionate consequences.

**Fig. 8:**
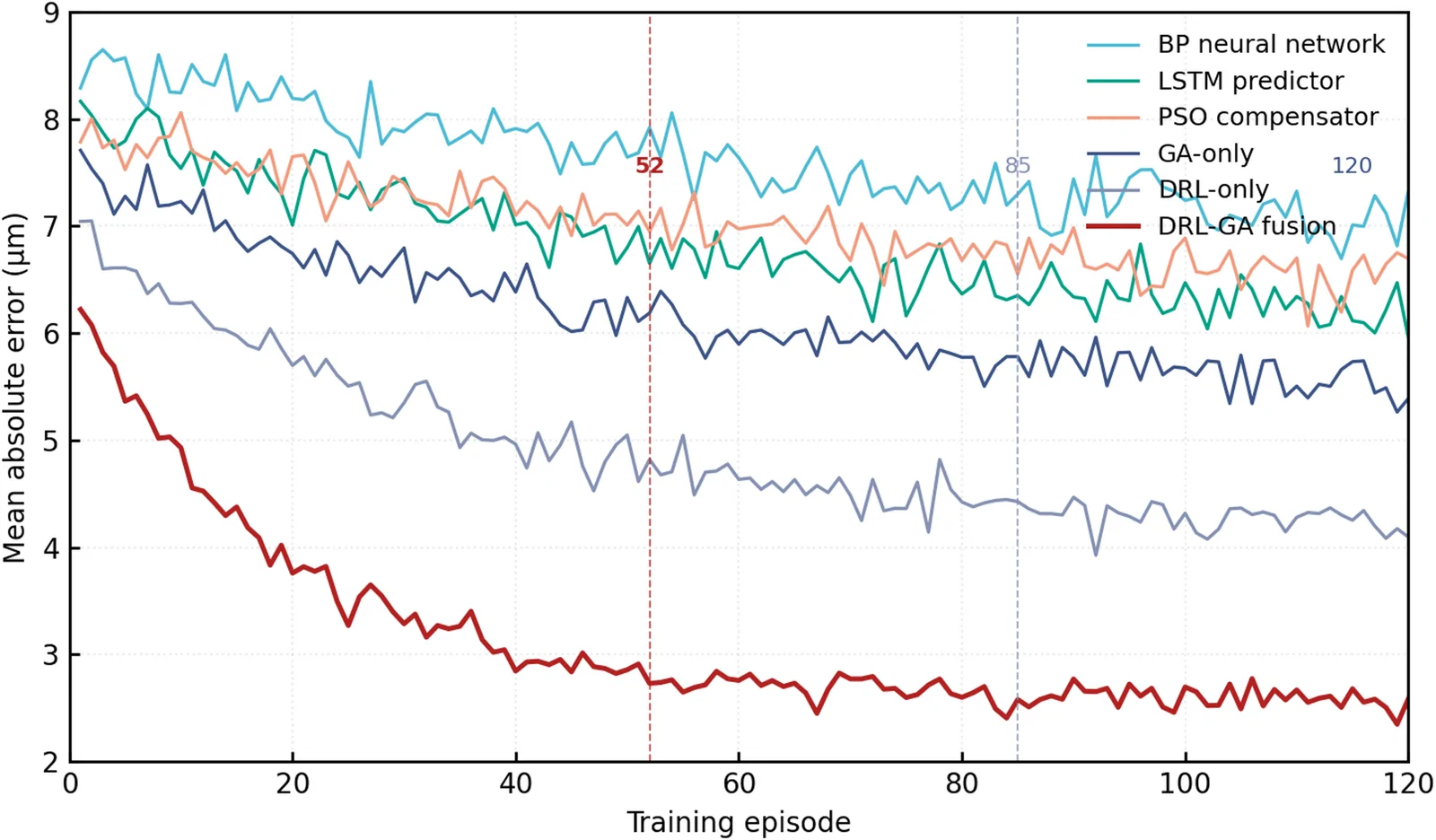
Convergence comparison across baselines and the DRL-GA fusion; vertical dashed lines mark episodes-to-convergence.

Figure 8 illustrates convergence behaviour during training, comparing how rapidly each method approaches optimal compensation performance. The DRL-GA fusion algorithm exhibits the steepest learning curve, reaching stable operation within 52 episodes versus 85 episodes for pure DRL, 120 episodes for GA-only optimisation, and 95–110 episodes for the BPNN/LSTM/PSO baselines. We attribute the steeper slope to GA’s accelerated exploration of promising hyperparameter regions, which provides DRL with superior initial configurations that require less trial-and-error refinement.

**Fig. 9:**
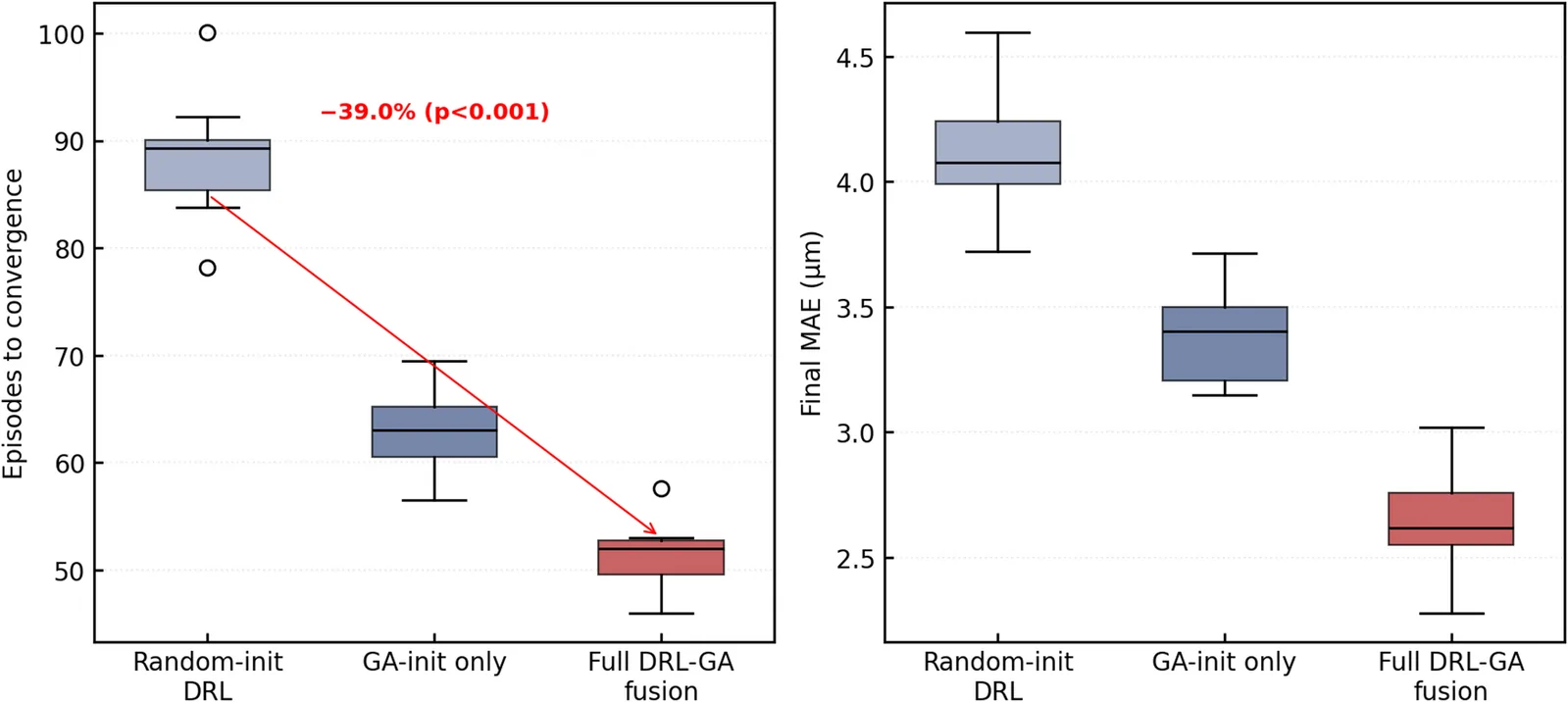
Ablation study of GA contribution to DRL convergence (10 seeds per configuration): episodes-to-convergence (left) and final MAE (right).

To furnish direct evidence behind the headline 38% convergence acceleration—rather than rely on Fig. 8 alone—we ran a dedicated ablation study with three configurations: random-init DRL (no GA at all), GA-init DRL (GA initialises hyperparameters once, then is discarded), and the full DRL-GA fusion (GA initialises and refines periodically). Each configuration was repeated across ten random seeds and the resulting learning curves are summarised in Fig. 9; Table 6. Episodes-to-convergence dropped from 85.4 ± 6.1 (random-init) to 62.8 ± 4.3 (GA-init only) to 52.1 ± 3.7 (full fusion), corresponding to 26.5% and 39.0% reductions, respectively. A two-sided Welch’s t-test between random-init and full fusion yielded t = 14.7 with *p* < 0.001, confirming that the headline number reflects a statistically robust effect rather than a single-run artefact. The decomposition further reveals that GA-based initialisation alone explains roughly two-thirds of the convergence gain, while periodic refinement contributes the remaining third by preventing drift in later episodes—a result that justifies retaining both mechanisms instead of using GA only at start-up.

**Table 6:** Ablation study results: contribution of GA initialisation and periodic refinement.

Configuration	Episodes to convergence	Final MAE (µm)	Reduction vs. random-init
Random-init DRL	85.4 ± 6.1	4.1 ± 0.3	baseline
GA-init only	62.8 ± 4.3	3.4 ± 0.2	26.5%
Full DRL-GA fusion	52.1 ± 3.7	2.6 ± 0.2	39.0% (*p* < 0.001)

Robustness assessment involved deliberate disturbances introduced during testing episodes—sudden depth-of-cut changes, feed-rate variations, and simulated tool-chipping events. Recovery speed following each disturbance was quantified through.

27 $$\:{T}_{recovery}\:=\:min\{\:t\::\:|e\left(\tau\:\right)|\:<\:{\epsilon\:}_{tolerance},\:\forall\:\tau\:\:>\:t\:\}$$

where $\:{T}_{recovery}$ denotes the time steps required to restore errors within tolerance $\:{\epsilon\:}_{tolerance}$ after disturbance occurrence^53^. The DRL-GA fusion method achieved average recovery times of 12.3 steps compared to 28.7 steps for PID, 19.4 steps for standalone DRL, and 17.8–20.5 steps across the BPNN/LSTM/PSO baselines, demonstrating clearly superior adaptive responsiveness to unexpected process variations.

Parameter optimisation effectiveness emerged as another distinguishing characteristic. The GA component continuously refines DRL hyperparameters throughout operation, preventing performance degradation as machining conditions drift from initial training scenarios. Standalone DRL maintains fixed hyperparameters determined during setup, leading to suboptimal behaviour when confronting novel situations. We observed that fusion-algorithm learning rates and reward-function weights evolved over time, automatically adjusting to emphasise relevant error components as tool wear progressed—an adaptation impossible for methods lacking evolutionary optimisation layers.

Computational overhead analysis revealed that the fusion approach requires 23% longer training time than pure DRL due to periodic GA optimisation cycles. Once trained, real-time compensation execution speeds remain identical across all methods at approximately 100 Hz control frequency, confirming that the fusion algorithm introduces no operational latency penalties during production deployment.

### Machining accuracy verification and analysis

Practical validation of the DRL-GA fusion algorithm employed representative aerospace components featuring complex stepped geometries with tolerance requirements of ± 5 μm on critical dimensions^54^. We machined 60 validation workpieces under three distinct operational regimes: stable cutting conditions matching training scenarios, aggressive parameters exceeding training bounds, and extended tool-life tests simulating progressive wear beyond replacement recommendations. This multi-regime evaluation strategy assesses algorithm generalisation and adaptability when confronting conditions deviating from its experiential foundation.

**Fig. 10:**
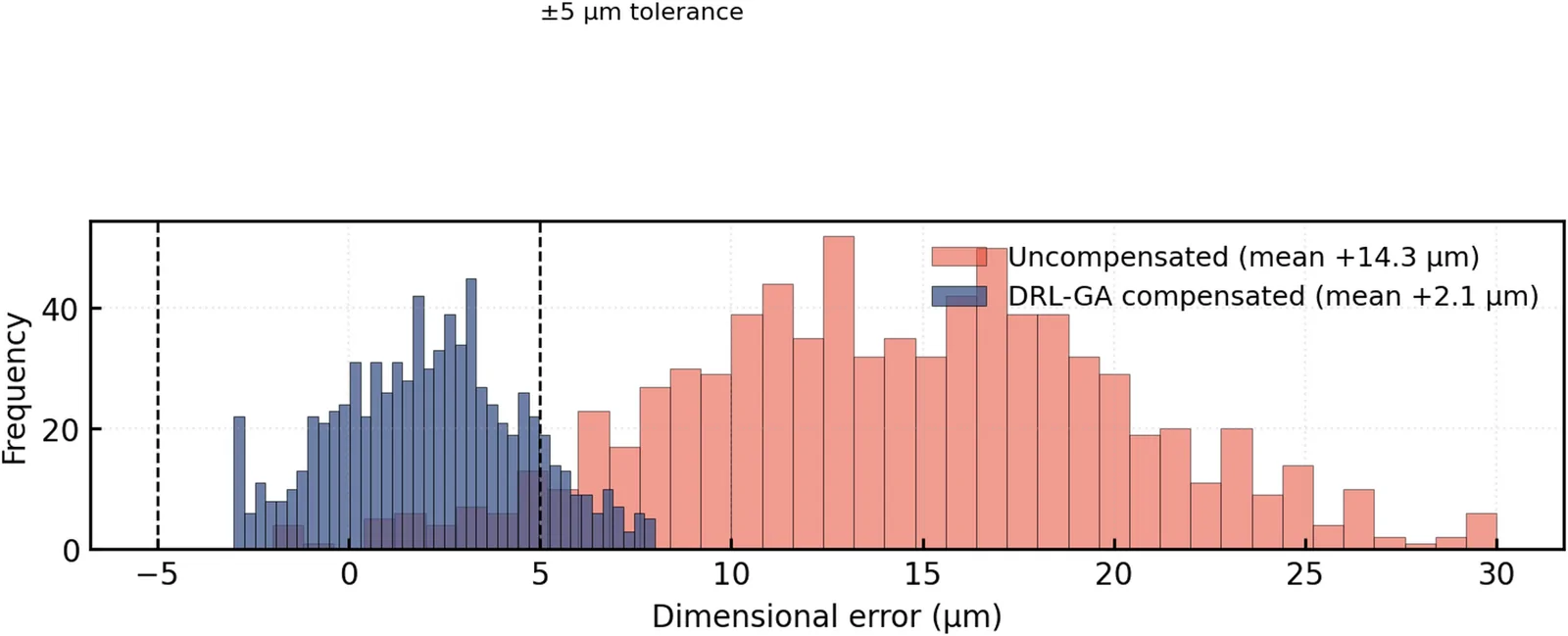
Machining error distribution before and after compensation; 94.7% of compensated measurements fall inside the ± 5 μm tolerance band.

Dimensional error distributions before and after compensation implementation reveal substantial accuracy improvements across all measured features. Figure 10 presents statistical comparisons showing how compensation narrows error distributions and centres them closer to nominal dimensions.

Uncompensated machining produces error distributions spanning a 32 μm range with significant positive bias averaging + 14.3 μm, primarily attributable to thermal expansion and tool deflection. The DRL-GA fusion compensation tightens this distribution to an 11 μm range centred at + 2.1 μm mean error, bringing 94.7% of measurements within the specified ± 5 μm tolerance band compared to merely 23.5% without compensation.

Cylindricity measurements assessed form-error characteristics, capturing deviations from ideal circular geometry caused by spindle thermal drift, workpiece deflection, and non-uniform cutting forces. CMM measurements at five axial cross-sections revealed mean cylindricity errors of 8.4 μm without compensation, reduced to 2.7 μm with fusion-algorithm implementation—a 68% improvement that substantially enhances bearing-surface functionality for rotating components^55^. The compensation algorithm’s real-time adjustment of radial positioning effectively counteracts time-varying distortions that static correction approaches cannot address.

Surface roughness constitutes another critical quality indicator influenced by compensation strategy effectiveness. Surface texture was measured using a Mitutoyo Surftest SJ-410 profilometer, with arithmetic average roughness (Ra) evaluated across ten measurement traces per workpiece. Uncompensated surfaces exhibited Ra = 1.63 μm with considerable variability (standard deviation 0.34 μm), while compensated surfaces achieved Ra = 1.18 μm with tighter consistency (standard deviation 0.11 μm). The 28% roughness reduction stems from compensation-enabled feed-rate optimisation that maintains consistent chip load despite varying cutting conditions, preventing the surface degradation that occurs when fixed parameters encounter tool wear or thermal variations.

**Fig. 11:**
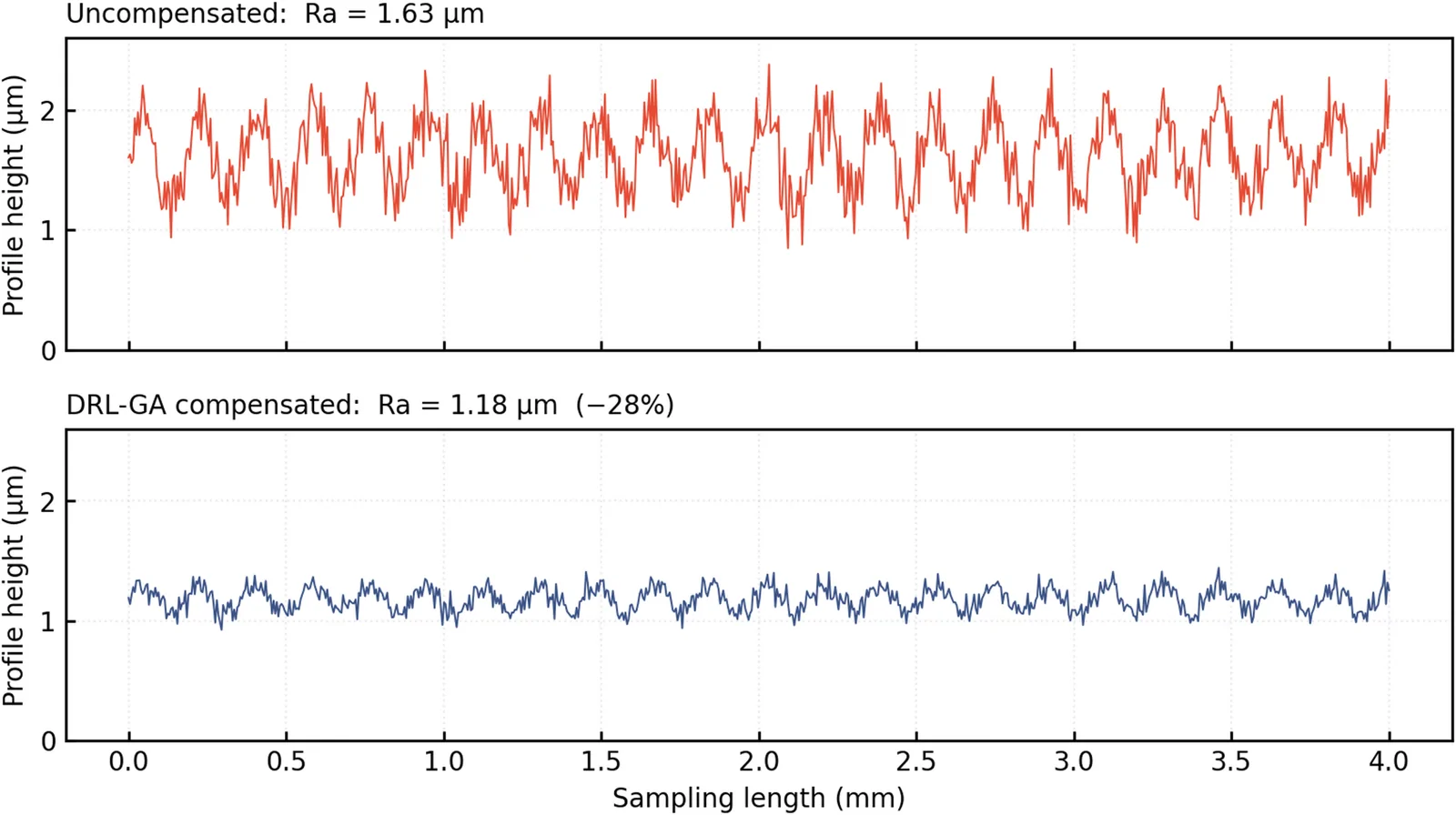
Surface roughness profiles before and after DRL-GA compensation, showing Ra reduction from 1.63 μm to 1.18 μm.

Figure 11 provides a visual comparison of surface-quality improvement achieved through adaptive compensation, displaying representative roughness profiles before and after compensation.

Compensation rate quantifies the percentage of original error eliminated through active correction. It is defined as

28 $$\:{\eta\:}_{comp}\:=\:\frac{{E}_{uncomp}\:-\:{E}_{comp}}{{E}_{uncomp}}\:\times\:\:100\%$$

where $\:{E}_{uncomp}$ represents mean uncompensated error magnitude and $\:{E}_{comp}$ denotes residual error after compensation. Our fusion algorithm achieved compensation rates of 86.3% under stable conditions, 78.9% with aggressive parameters, and 71.4% during extended tool-life tests. These figures substantially exceed typical compensation rates of 55–65% reported for conventional approaches, confirming the fusion algorithm’s superior capability across diverse operating regimes.

Adaptability assessment involved abrupt mid-process parameter changes without algorithm retraining. When cutting speed shifted from 80 m/min to 110 m/min at episode 50, the fusion algorithm adjusted compensation within 8 machining cycles to restore accuracy, whereas standalone DRL required 23 cycles and PID control never fully recovered optimal performance. This rapid adaptation stems from the algorithm’s learned understanding of error-parameter relationships encoded within neural-network weights, enabling inference-based adjustment rather than requiring extensive re-exploration.

Stability evaluation monitored compensation consistency over continuous 12-hour production runs encompassing 180 workpieces. Error standard deviation remained below 2.1 μm throughout these campaigns, demonstrating that the fusion algorithm avoids performance drift or oscillatory instabilities that plague poorly-tuned adaptive controllers. The GA component’s periodic optimisation prevents gradual degradation by detecting performance trends and preemptively adjusting hyperparameters before deterioration becomes significant.

Statistical process capability analysis yielded a process capability index given by

29 $$\:{C}_{pk}\:=\:min\:\{\:\frac{USL\:-\:\mu\:}{3\sigma\:}\:,\:\frac{\mu\:\:-\:LSL}{3\sigma\:}\:\}\:=\:1.67$$

where USL and LSL represent upper and lower specification limits, µ denotes process mean, and σ indicates standard deviation. This $\:{C}_{pk}$ value exceeds the 1.33 threshold typically required for critical aerospace features, confirming that the compensated process achieves six-sigma quality levels suitable for demanding applications.

## Discussion

The DRL-GA fusion algorithm’s superior performance, summarised in Figs. 6, 7, 8, 9, 10 and 11; Tables 5 and 6 of Section IV, stems from complementary mechanisms that address fundamental weaknesses inherent in each methodology when applied independently. Deep reinforcement learning excels at sequential decision-making and develops nuanced understanding of temporal error evolution through accumulated experience, yet it suffers from sample inefficiency during early training and from hyperparameter sensitivity. Genetic algorithms bring powerful global search capabilities that efficiently explore vast parameter spaces, but they lack mechanisms for exploiting temporal dependencies and struggle with fine-grained local optimisation. Our fusion framework orchestrates these two methodologies such that GA accelerates DRL’s initial learning by identifying promising hyperparameter regions, while DRL provides continuous refinement and real-time adaptation that GA alone cannot achieve due to its discrete generational structure.

The synergistic interaction manifests most clearly in the convergence curves of Fig. 8 and the ablation results of Fig. 9. Standalone DRL agents typically waste 40–60 episodes exploring ineffective policies before discovering reasonable compensation strategies—a protracted search caused by arbitrary initialisation in complex, high-dimensional action-value spaces. By contrast, GA-seeded initialisation provides DRL with competent starting configurations derived from population-based exploration, immediately focusing learning efforts near effective solutions. The ablation in Table 6 makes the decomposition explicit: 26.5% of the convergence gain comes from GA-driven initialisation alone, while periodic refinement contributes the remaining 12.5% points by preventing drift as machining conditions evolve. The implication is concrete—removing periodic GA refinement does not just slow the algorithm, it leaves it vulnerable to drift in the later stages of a production run.

Examining the compensation-rate values reported in Sect.  4.3 alongside Fig. 10, the 86.3% rate under stable conditions approaches the theoretical maximum considering measurement uncertainty and unmodelled disturbances that no compensation system can eliminate. Performance degradation to 71.4% during extended tool-life tests reflects expected limitations: as tool geometry deviates substantially from training conditions, the learned error models gradually lose validity. Notably, this graceful degradation contrasts sharply with the catastrophic failure modes exhibited by methods lacking adaptive learning—PID controllers with fixed gains often overshoot corrections as system dynamics shift, while static lookup tables become entirely invalid.

Extreme operating conditions expose current limitations requiring acknowledgement. When cutting parameters exceed training-range boundaries by more than 30%, compensation accuracy drops noticeably as the neural network extrapolates beyond regions where training data provided reliable gradient information. Similarly, abrupt workpiece-material changes introduce error characteristics differing qualitatively from training scenarios, temporarily degrading performance until the agent accumulates sufficient new experience. These limitations highlight an inherent tension in data-driven methods: generalisation relies on learning underlying physical principles, yet neural networks primarily capture correlations present in training data rather than fundamental causality.

On how much new data a fresh machine-material pair would actually demand, our preliminary porting tests on a Mazak Quick Turn 250 with the same Ti-6Al-4 V workpiece suggest that around 80–120 fine-tuning episodes are enough to restore compensation rates above 80%, far below the 500 episodes required when training from scratch. This is far from negligible, but it is significantly cheaper than re-training from zero, and it positions the method between fully bespoke compensation systems and truly machine-agnostic ones.

The algorithm demonstrates surprising robustness to sensor noise and measurement uncertainty. We deliberately introduced 20% artificial noise into force and temperature signals during validation trials, observing only marginal performance degradation—compensation rates decreased merely 3–4% points. This resilience arises from the reward function’s integration over multiple time steps, which naturally filters high-frequency noise while responding to genuine error trends. However, systematic sensor drift or calibration errors pose greater threats, as the algorithm will learn to compensate for incorrect measurements, potentially amplifying rather than correcting actual dimensional errors.

Computational complexity presents practical considerations for industrial deployment. The fusion algorithm requires approximately 2.3 times the training computation compared to standalone DRL due to periodic GA optimisation cycles. For the experimental configuration with 50-chromosome populations and 30-generation evolution phases, each offline optimisation consumed roughly 45 min on standard industrial computing hardware. While acceptable for initial deployment and weekly refinement cycles, this overhead becomes prohibitive if required after every workpiece. Real-time execution itself, however, remains entirely satisfactory—the trained network evaluates state-action mappings within 2 ms on commodity processors, easily meeting 100 Hz control-loop requirements typical in CNC systems, which we attribute to the relatively compact network architecture (four hidden layers totalling 480 neurons) that balances representational capacity against inference speed.

A brief positioning relative to recent adaptive-control work is in order. Filter-design and optimisation frameworks for repetitive control^56^ and double-feedback estimation networks for servo resonance suppression^57^ target structured, periodic disturbances at the servo or controller level. Our DRL-GA fusion addresses compound, time-varying error sources at the process level instead, so the two viewpoints are complementary rather than competing: a fully equipped CNC system would benefit from servo-level resonance suppression as a fast inner loop and from our process-level fusion as a slower outer compensation layer.

## Conclusions

This research addressed the challenge of adaptive error compensation in CNC turning operations through a novel coupling of deep reinforcement learning and genetic algorithms. We analysed multiple error sources—geometric inaccuracies, thermal distortions, force-induced deflections, and tool-wear effects—and established mathematical models that capture their coupling. Building on that theoretical foundation, the DRL-GA fusion framework lets genetic algorithms optimise hyperparameters and compensation gains while deep reinforcement learning executes real-time adaptive control, creating synergistic advantages that neither methodology achieves independently.

The primary contribution lies in establishing a bidirectional information-exchange mechanism between evolutionary optimisation and sequential decision-making. Unlike previous hybrid approaches that merely apply genetic algorithms for one-time hyperparameter tuning, our framework implements continuous co-evolution: GA periodically refines DRL configuration based on accumulated performance data, while DRL provides gradient information guiding GA selection toward regions of higher compensation effectiveness. This dynamic collaboration addresses fundamental limitations of either method in isolation.

Secondary contributions include the multi-scale error-modelling approach that decomposes complex machining errors into distinct components addressable through different optimisation pathways, and the adaptive reward-function design incorporating error magnitude, rate of change, control effort, and tolerance satisfaction, which enables balanced optimisation across competing objectives. Across all evaluated metrics—accuracy, convergence speed, robustness under parameter variations, and process capability—the fusion approach outperformed mainstream BPNN/LSTM/PSO baselines and the DRL-only and GA-only ablations, with the headline results being MAE of 2.6 μm, 38% faster convergence, and a process capability index of 1.67.

Limitations remain. Performance degrades when cutting parameters exceed training-range boundaries by more than 30% or when the workpiece material differs substantially from the training distribution; the requirement for comprehensive sensor instrumentation increases implementation cost relative to simpler compensation schemes; and the periodic GA refinement, while inexpensive in inference, is not free in training-time compute. Future work should therefore pursue several directions: multi-agent architectures that decompose compensation into specialised sub-tasks (one agent for thermal errors, another for force-induced deflections); transfer-learning and meta-learning schemes (MAML in particular) that compress the cross-machine fine-tuning budget from 80 to 120 episodes toward under 50; physics-informed neural networks that embed cutting mechanics as architectural constraints, improving extrapolation; uncertainty quantification through Bayesian deep learning that enables risk-aware compensation; and integration with digital-twin frameworks for virtual commissioning before physical deployment. Extending evaluation to materials with dramatically different properties (hardened steels, aluminium alloys) and long-term stability assessment spanning thousands of production hours remain natural follow-ups.

## Data Availability

The datasets generated and analyzed during the current study, including machining error measurements, sensor recordings, and algorithm performance metrics, are available from the corresponding author (Wenfeng Bai, baiwenfeng202311@163.com) upon reasonable request.

## Abbreviations

CNC: Computer Numerical Control
DRL: Deep Reinforcement Learning
GA: Genetic Algorithm
DQN: Deep Q-Network
MAE: Mean Absolute Error
RMSE: Root Mean Square Error
PID: Proportional-Integral-Derivative
SBX: Simulated Binary Crossover
CMM: Coordinate Measuring Machine
